# Partial Restoration of Mutant Enzyme Homeostasis in Three Distinct Lysosomal Storage Disease Cell Lines by Altering Calcium Homeostasis

**DOI:** 10.1371/journal.pbio.0060026

**Published:** 2008-02-05

**Authors:** Ting-Wei Mu, Douglas M Fowler, Jeffery W Kelly

**Affiliations:** Department of Chemistry and The Skaggs Institute for Chemical Biology, The Scripps Research Institute, La Jolla, California, United States of America; University of California, San Francisco, United States of America

## Abstract

A lysosomal storage disease (LSD) results from deficient lysosomal enzyme activity, thus the substrate of the mutant enzyme accumulates in the lysosome, leading to pathology. In many but not all LSDs, the clinically most important mutations compromise the cellular folding of the enzyme, subjecting it to endoplasmic reticulum–associated degradation instead of proper folding and lysosomal trafficking. A small molecule that restores partial mutant enzyme folding, trafficking, and activity would be highly desirable, particularly if one molecule could ameliorate multiple distinct LSDs by virtue of its mechanism of action. Inhibition of L-type Ca^2+^ channels, using either diltiazem or verapamil—both US Food and Drug Administration–approved hypertension drugs—partially restores N370S and L444P glucocerebrosidase homeostasis in Gaucher patient–derived fibroblasts; the latter mutation is associated with refractory neuropathic disease. Diltiazem structure-activity studies suggest that it is its Ca^2+^ channel blocker activity that enhances the capacity of the endoplasmic reticulum to fold misfolding-prone proteins, likely by modest up-regulation of a subset of molecular chaperones, including BiP and Hsp40. Importantly, diltiazem and verapamil also partially restore mutant enzyme homeostasis in two other distinct LSDs involving enzymes essential for glycoprotein and heparan sulfate degradation, namely α-mannosidosis and type IIIA mucopolysaccharidosis, respectively. Manipulation of calcium homeostasis may represent a general strategy to restore protein homeostasis in multiple LSDs. However, further efforts are required to demonstrate clinical utility and safety.

## Author Summary

Lysosomes are organelles that contain more than 50 hydrolytic enzymes that break down macromolecules in a cell. A lysosomal storage disease results from deficient activity of one or more of these enzymes, leading to the accumulation of corresponding substrate(s). Currently, lysosomal storage diseases are treated by enzyme replacement therapy, which can be challenging because the enzyme has to enter the cell and the lysosome to function; in neuropathic diseases, enzyme replacement is not useful because recombinant enzymes do not enter the brain. We have shown that diltiazem and verapamil, potent US Food and Drug Administration–approved L-type Ca^2+^ channel blocker drugs, increased the endoplasmic reticulum (ER) folding capacity, trafficking, and activity of mutant lysosomal enzymes associated with three distinct lysosomal storage diseases. These compounds appear to function through a Ca^2+^ ion–mediated up-regulation of a subset of cytoplasmic and ER lumenal chaperones, possibly by activating signaling pathways that mitigate cellular stress. We have shown that increasing ER calcium levels appears to be a relatively selective strategy to partially restore mutant lysosomal enzyme homeostasis in diseases caused by the misfolding and degradation of nonhomologous mutant enzymes. Because diltiazem crosses the blood–brain barrier, it may be useful for the treatment of neuropathic lysosomal storage diseases, and possibly other loss-of-function diseases, although efficacy needs to be demonstrated.

## Abstract 3

By adapting the protein homeostasis network, altering calcium homeostasis can restore the cell's ability to fold and traffic proteins prone to misfolding, offering a new strategy to ameliorate loss-of-function diseases.

## Introduction

Cells usually maintain a balance between protein synthesis, folding, trafficking, aggregation, and degradation—which is referred to as protein homeostasis—by using sensors and networks of pathways [1,2]. Human loss-of-function diseases are often the result of a disruption of normal protein homeostasis, which is typically caused by a mutation in a given protein that compromises its cellular folding, leading to efficient degradation [3]. Thus, there is insufficient function, because the concentration of the mutant protein is exceedingly low.

There are at least 40 distinct lysosomal storage diseases (LSDs) resulting from the deficient function of a single mutated enzyme in the lysosome, leading to accumulation of corresponding substrate(s) [4,5]. Currently, LSDs are treated by enzyme replacement therapy, which can be challenging because the endocytic system has to be used to get the recombinant enzyme into the lysosome [6].

The most prevalent LSD is Gaucher disease (GD), which is caused by a deficiency in the activity of lysosomal glucocerebrosidase (GC), a glycolipid hydrolase [7]. Glucosylceramide accumulation in Gaucher monocyte-macrophage cells leads to hepatomegaly, splenomegaly, anemia, thrombocytopenia, bone lesions, and in severe cases, central nervous system (CNS) involvement [8]. Patients without CNS involvement are classified as type I (mild adult onset), whereas those with CNS involvement are classified as type II (acute infantile onset) or type III (subacute juvenile or early adult onset). The clinically most important GC mutations, such as N370S, the most common mutation associated with type I GD, and L444P, the most prevalent mutation resulting in CNS involvement, predispose GC to misfold in the endoplasmic reticulum (ER), subjecting these variants to ER-associated degradation (ERAD), reducing the normal amount of mutant GC trafficking to the lysosome. Thus, the mutant GC concentration in the lysosome is substantially reduced [9,10]. Many of the folding-deficient GC variants exhibit fractional specific activity when properly folded [11], demonstrating that if folding and trafficking of the mutated enzymes could be enhanced, it is likely that the disease would be ameliorated.

The US Food and Drug Adminidtration (FDA) has approved enzyme replacement therapy and substrate reduction therapy to treat type I GD [5,12]. There is currently no effective treatment for neuropathic GD (types II and III); the recombinant GC enzyme does not cross the blood–brain barrier and the efficacy of the substrate reduction drug in the CNS remains unclear, hence a novel strategy for neuropathic GD would be welcomed. Pharmacological chaperoning is an emerging therapeutic strategy that uses ER-permeable small molecules that bind to and stabilize the folded state of a given enzyme, enabling its trafficking to the Golgi and onward to the lysosome [13–23]. Whereas patient-derived cells harboring most GD-associated mutations appear to be amenable to pharmacological chaperoning, cell lines harboring the L444P GC mutation have thus far proven refractory, although alternative dosing regimens could ultimately be useful [17].

α-Mannosidosis and type IIIA mucopolysaccharidosis (MPS) are neuropathic LSDs caused by the inability of the lysosome to degrade glycoproteins and heparan sulfate, respectively [5,24,25]. The P356R mutation in lysosomal α -mannosidase alters the folding energy landscape resulting in severe infantile α-mannosidosis associated with rapid mental deterioration [26]. The prevalent S66W or R245H heparan sulfate sulfamidase (SGSH) mutations in type IIIA MPS reduce mutant enzyme concentrations in the lysosome, most likely due to impaired folding and ERAD in lieu of efficient folding and trafficking of sulfamidase, leading to the accumulation of heparan sulfate and severe CNS degeneration [27]. Currently no effective therapy is available for α-mannosidosis or type IIIA MPS, hence new strategies for these neuropathic LSDs would be welcomed.

The Ca^2+^ ion is a universal and extremely important signaling ion in the cell. Ca^2+^ signaling affects numerous cellular functions by diverse pathways and is a primary regulator of ER function [28–30]. Emerging evidence indicates that calcium signaling may influence diseases associated with deficiencies in protein homeostasis, including many LSDs [4,31,32]. This hypothesis is supported by observations that manipulation of calcium homeostasis by sarcoplasmic endoplasmic reticulum Ca^2+^-ATPase (SERCA) inhibitors, such as thapsigargin [33] and curcumin [34], enhances folding and trafficking of the ΔF508 cystic fibrosis transmembrane conductance regulator (CFTR).

The hypothesis that manipulation of intracellular calcium homeostasis might improve defects in mutant enzyme homeostasis that lead to LSDs was scrutinized. Small molecules were sought that enhance the folding, trafficking, and function of endogenous mutant lysosomal enzymes in multiple cell lines associated with different LSDs, thus restoring function by repairing instead of replacing the damaged enzyme through altering calcium homeostasis. In this quest, we discovered that the FDA-approved drugs diltiazem and verapamil, both L-type voltage-gated calcium channel blockers [35], partially restored mutant lysosomal enzyme function in three distinct LSDs caused by folding defects in nonhomologous enzymes. These results suggest that calcium channel blockers are promising candidates to enhance lysosomal enzyme homeostasis in a variety of LSDs, although more investigation is necessary to demonstrate clinical utility and safety.

## Results

### The L-type Ca^2+^ Channel Blockers Diltiazem and Verapamil Enhance Lysosomal GC Activity in Gaucher Patient-Derived Fibroblast Cell Lines

**Figure 1:**
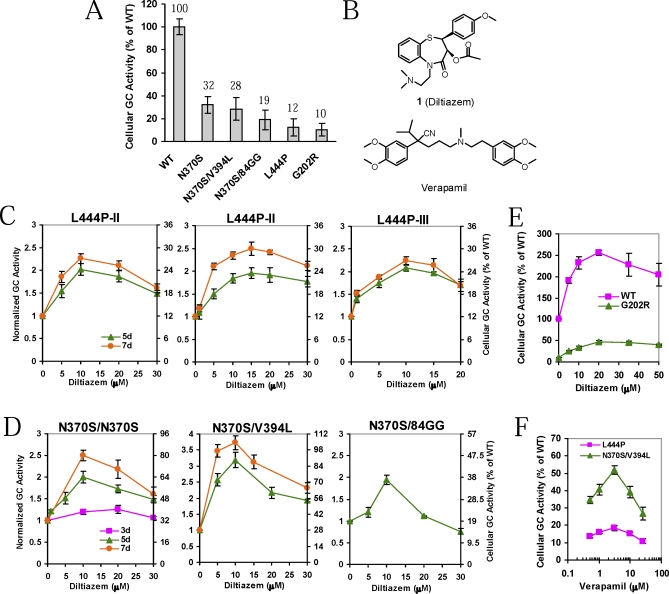
Influence of Small Molecules on GC Variant Activity in Gaucher Patient-Derived Fibroblasts (A) Residual activities of GC variants using the lysed cell GC activity assay, using equal numbers of cells as ascertained from the equal total protein content of the lysate. Residual activities of N370S, N370S/V394L, N370S/84GG, L444P, and G202R GC are shown as the percentage of WT GC activity (numbers above each column). (B) Chemical structures of diltiazem (compound **1**) and verapamil. (C) The influence of diltiazem (**1**) on L444P GC activity in three distinct homozygous L444P GC patient-derived cell lines: L444P GC fibroblasts from a type II patient (left panel), another type II patient (middle panel), and a type III patient (right panel). These cell lines were cultured with diltiazem for 5 d (green line) and 7 d (orange line). The GC activity of treated cells was normalized against that of untreated cells of the same type (left *y*-axis) and expressed as the percentage of WT GC activity (right *y*-axis). (D) The influence of diltiazem on N370S GC activity in homozygous N370S/N370S GC fibroblasts (left panel), heterozygous N370S/V394L fibroblasts (middle panel), and heterozygous N370S/84GG fibroblasts (right panel). These cell lines were cultured with diltiazem for 3 d (pink line), 5 d (green line), and 7 d (orange line). The GC activity of treated cells was normalized against that of untreated cells of the same type (left *y*-axis) and expressed as the percentage of WT GC activity (right *y*-axis). (E) The influence of diltiazem on WT and G202R GC activity. WT and G202R GC cell lines were cultured with diltiazem for 5 d. The GC activity was expressed as the percentage of WT GC activity. (F) The influence of verapamil on L444P and N370S/V394L GC activity. These cell lines were treated with verapamil for 7 d. The GC activity was expressed as the percentage of WT GC activity.

Several type I, II, and III Gaucher fibroblast cell lines were evaluated to discern the relative activity of the mutant GC harbored by equal numbers of cells reflected by an equal amount of total protein in the cell lysate. The residual enzymatic activities of the GC variants were measured using the lysed cell activity assay (Figure 1A). Under the assay conditions used, L444P GC exhibits 12% of the activity of wild-type (WT) GC; N370S GC, 32%; N370S/V394L GC, 28%; N370S/84GG GC, 19%; and G202R GC, 10% of the activity of WT GC. The exact enzyme activity measured is highly dependent on the assay conditions [17]. The enzyme activities displayed were normalized to the activity of untreated cells of the same type and expressed as fold changes. In addition, mutant GC activity before and after treatment was also expressed as the percentage of WT GC activity to calibrate the reader.

Gaucher patient–derived fibroblasts harboring the L444P GC mutation grown at 30 °C, instead of at 37 °C, exhibit enhanced folding, trafficking, lysosomal localization, and activity of L444P GC [10]. Because a change in temperature alters both the physical chemistry of the L444P protein and the cellular protein homeostasis machinery, the hypothesis that cells were readjusting their protein homeostasis capacity through their thermosensitive transient receptor potential (TRP) channels was tested. Fifteen thermosensitive TRP channel modulators (capsaicin, resiniferatoxin, piperine, olvanil, anandamide, 2-APB, camphor, 4α-PDD, menthol, eucalyptol, icilin, cinnamaldehyde, allylisothiocyanate, capsazepine, and ruthenium red) were administered to homozygous L444P GC patient–derived fibroblasts [36,37]. Enhanced L444P GC folding and trafficking could be inferred from increased lysosomal L444P GC activity measured using the intact cell GC enzyme assay. Only ruthenium red notably increased L444P GC activity after a 5-d incubation period (Figure S1). Because the only TRP channel modulator that enhanced lysosomal L444P GC activity was also a nonspecific Ca^2+^ channel blocker, the observed increase might be a consequence of a lowered intracellular Ca^2+^ ion concentration, and thus it seemed unlikely that TRP channel modulation is the means by which temperature regulates the intracellular protein homeostasis capacity.

The hypothesis that ruthenium red was lowering the intracellular Ca^2+^ ion concentration and enhancing GC folding fidelity by that means was scrutinized experimentally by blocking calcium entry into the cell through its calcium channels, including voltage-gated calcium channels (VGCCs) and ionotropic glutamate receptors [28,38,39]. Lysosomal L444P GC activity was evaluated after the separate application of nine representative VGCC blockers—namely diltiazem, verapamil, nifedipine, nimodipine, loperamide, mibefradil, ethosuximide, flunarizine, and bepridil—five representative ionotropic glutamate receptor inhibitors—namely CGP 39551, 5,7-dichlorokynurenic acid, DNQX, Evans blue, and felbamate—and other calcium channel blockers—such as amiodarone, cinnarizine, and SKF 96365—to L444P GC patient-derived fibroblasts. The intact cell GC activity assay revealed that only diltiazem and verapamil (chemical structures shown in Figure 1B) increased L444P GC activity significantly. Diltiazem increased L444P GC activity a maximum of 2.0-fold (to ≈24% of normal cellular WT GC activity) after a 5-d incubation period, and 2.3-fold after a 7-d incubation period (Figure 1C, left panel) at a 10 μM concentration (all concentrations mentioned are cell culture concentrations unless otherwise stated), implying an increased lysosomal L444P GC concentration. The temporal dependence of the diltiazem-mediated cellular L444P GC activity increase is very similar to the time dependence of the cellular N370S GC activity increase observed upon pharmacological chaperone treatment [17]. The slow gain in activity of the GC variants is partially a result of the slower folding and trafficking of the variants as revealed by prior pulse chase experiments [18,40] and partially for other reasons, including the apparent requirement for the transcription and translation of selected chaperones (vide infra). This slow mutant GC activity increase upon pharmacological chaperone or diltiazem administration is also observed upon pharmacological chaperone administration in different LSDs such as Fabry disease, Tay-Sachs disease, and Pompe disease [13,20,41].

To confirm that the effect of diltiazem was not restricted to one L444P GC patient-derived cell line, two additional patient-derived homozygous L444P GC fibroblast cell lines were treated with diltiazem. In a type II cell line, diltiazem (15 μM) increased the GC activity up to 2.0-fold after an incubation period of 5 d and up to 2.5-fold after 7 d of treatment (Figure 1C, middle panel). Diltiazem (10 μM) treatment of a type III Gaucher patient-derived cell line increased L444P GC activity to a maximum of 2.1-fold after a 5-d incubation period and up to 2.3-fold after a 7-d incubation period (Figure 1C, right panel). Lysosomal L444P GC activity was improved in all the neuropathic fibroblast cell lines evaluated.

If diltiazem regulates mutant GC homeostasis by a general mechanism, such as a cellular chaperone–mediated mechanism, and not by binding induced pharmacological chaperoning, it should also be able to enhance the folding, trafficking and activity of other misfolding-prone GC variants in homozygous and compound heterozygous Gaucher patient-derived cell lines. Diltiazem (10 μM) increased N370S GC activity up to 2.0-fold (to ≈64% of untreated WT GC activity) after a 5-d incubation period and up to 2.5-fold after a 7-d incubation period in N370S GC fibroblasts from a homozygote (Figure 1D, left panel), analogous to the best results obtained with optimized pharmacological chaperones [22]. In the case of a compound heterozygous N370S/V394L GC cell line, diltiazem (10 μM) increased the GC activity up to 3.2-fold (to ≈89% of cellular WT GC activity) after an incubation period of 5 d and up to 3.7-fold after 7 d of treatment (Figure 1D, middle panel). In the analogous N370S/84GG GC cell line, diltiazem (10 μM) increased the GC activity up to 1.9-fold (to ≈36% of cellular WT GC activity) after a 5-d treatment (Figure 1D, right panel). Diltiazem (20 μM) increased G202R GC activity up to 4.6-fold (to ≈46% of cellular WT GC activity) after a 5-d incubation period (Figure 1E, green line), demonstrating the generality of diltiazem to regulate GC protein homeostasis. Notably, diltiazem (20 μM) increases WT GC activity up to 2.6-fold after 5 d of treatment (Figure 1E, pink line), suggesting that the folding and trafficking of WT GC is inefficient, as is the case for other proteins such as G-protein-coupled receptors [42] and ion channels [43].

The influence of diltiazem on the cellular activity of other WT lysosomal hydrolases, namely α-mannosidase, α-glucosidase, β-galactosidase, α-galactosidase, and β-glucuronidase was evaluated. WT fibroblasts and L444P GC fibroblasts were incubated with diltiazem (10 μM) for 5 d before the analysis (Figure S2). While diltiazem treatment increased GC activity, it did not significantly increase the activity of other WT lysosomal enzymes, implying that the folding and trafficking of these enzymes is near optimal.

A second L-type Ca^2+^ channel blocker, verapamil (3 μM), increased L444P GC activity up to 1.5-fold (to ≈18% of cellular WT GC activity) and N370S/V394L GC activity up to 1.9-fold (to ≈53% of cellular WT GC activity) after a 7-d incubation period (Figure 1F; lysed cell activity assay). That both diltiazem and verapamil, which are Ca^2+^ channel blockers with distinct chemical structures (Figure 1B), enhance cellular mutant GC folding, trafficking, and activity supports the hypothesis that altering intracellular Ca^2+^ homeostasis influences lysosomal enzyme homeostasis.

### GC Exhibits a Dose-Dependent Concentration Increase Upon Diltiazem Treatment

**Figure 2:**
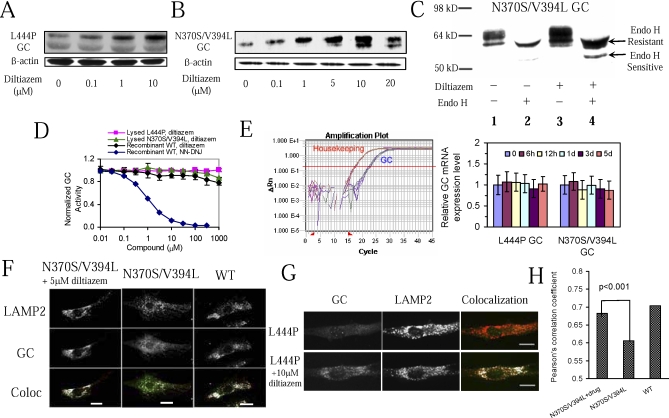
Effect of Diltiazem on L444P and N370S/V394L GC Folding and Trafficking (A) Western blot analysis of untreated and diltiazem-treated L444P GC cells. L444P GC cells were cultured without or with diltiazem at varying concentrations for 7 d before the cells were lysed for SDS-PAGE and Western blot analysis. GC was detected using mouse anti-GC antibody 2E2. β-actin served as a loading control. (B) Western blot of untreated and diltiazem-treated N370S/V394L GC cells. N370S/V394L GC cells were incubated with variable diltiazem concentrations for 7 d before the cells were lysed for SDS-PAGE and Western blot analysis using mouse anti-GC antibody 8E4. (C) The endo-H sensitivity of untreated and diltiazem-treated N370S/V394L GC cells. N370S/V394L GC cells were incubated without and with 10 μM diltiazem for 7 d before the cells were lysed for endo-H digestion, SDS-PAGE, and Western blot analysis using mouse anti-GC antibody 8E4. (D) L444P and N370S/V394L GC cells were lysed and an equal amount of total cell protein was incubated with diltiazem and their GC activities were evaluated using the lysed cell GC activity assay. Cerezyme, a recombinant WT GC protein, was also tested for its GC activity after treatment with diltiazem (black line) or a pharmacological chaperone NN-DNJ, a known inhibitor (blue line). (E) Quantitative RT-PCR on untreated and diltiazem-treated L444P and N370S/V394L GC cells. L444P and N370S/V394L GC cells were incubated with 10 μM diltiazem for 6 h, 12 h, 1 d, 3 d, and 5 d. The figure on the left is the representative amplification plot for the quantitative PCR cycles using L444P GC cells; the figure on the right shows the relative GC mRNA expression level for diltiazem-treated L444P (left entries) and N370S/V394L GC cells (right entries), respectively, which is normalized to that of untreated cells. (F) Immunofluorescence colocalization analysis of GC in N370S/V394L GC and WT fibroblasts. N370S/V394L GC cells were incubated with 5 μM diltiazem for 7 d (column 1) or cultured without drug (column 2). Untreated WT cells were observed as a positive control (column 3). GC was visualized using mouse anti-GC antibody 16B3 (row 2) and rabbit anti-LAMP2 antibody was applied as a lysosomal marker (row 1). In row 3, the colocalization of GC (green) and LAMP2 (red) is shown in white. Bar = 10 μm. (G) Immunofluorescence colocalization analysis of GC in L444P GC cells. L444P GC cells were incubated with 10 μM diltiazem for 14 d (bottom row) or untreated (top row). GC visualization was accomplished using the mouse anti-GC antibody 8E4 (column 1); rabbit anti-LAMP2 antibody was used as a lysosomal marker (column 2). In column 3, the colocalization of GC (green) and LAMP2 (red) is artificially colored white. Bar = 20 μm. (H) Quantification of the colocalization between the GC protein and the lysosomal marker using PCC. Experimental conditions were stated in Figure 2F.

Western blot analysis reveals that L444P GC concentrations were elevated in a dose-dependent manner in type II Gaucher fibroblasts after a 7-d treatment with diltiazem (Figure 2A). β-actin served to ensure that equal amounts of total protein were loaded in each lane. The GC band intensity increases with the concentration of added diltiazem (0, 0.1, 1, and 10 μM), consistent with the observed dose-dependent increase in GC enzymatic activity (Figure 1C, left panel).

The patient-derived N370S/V394L GC cell line was also cultured with diltiazem (0.1–10 μM) for 7 d, revealing an analogous dose-dependent increase in GC band intensity (Figure 2B), consistent with the concentration-dependent GC activity increase (Figure 1D, middle panel). An endo-H digestion was performed on treated and untreated N370S/V394L GC cells to demonstrate that the mature lysosomal glycoform of GC, associated with proper lyososmal trafficking, was being produced. After 7 d of cell culturing, equal numbers of diltiazem-treated (10 μM) and untreated cells (reflected by equal amounts of total protein) were subjected to endo-H treatment or buffer only treatment before separation on a 10% SDS-PAGE gel and detection of GC by Western blot analysis (Figure 2C). The upper bands in lanes 2 and 4 corresponding to the endo-H resistant, mature lysosomal GC glycoform increase upon diltiazem treatment [10], demonstrating that substantially more properly folded GC protein was trafficked out of the ER and to the lysosome after diltiazem treatment. The lower bands in lanes 2 and 4 correspond to the endo-H sensitive, ER GC glycoform.

### Ruling out a Pharmacological Chaperoning Mechanism and a Direct Lysosomal GC Activation Mechanism

All of the GC pharmacological chaperones discovered to date are active-site–directed stabilizers and are thus enzyme inhibitors; therefore we evaluated whether diltiazem binds to the active site and inhibits GC. Lysed L444P fibroblasts and lysed N370S/V394L cells were incubated with diltiazem (0.01 to 1000 μM) and assayed. No significant GC inhibition was observed in either case (Figure 2D, pink and green lines). Cerezyme, a recombinant version of WT GC, was also incubated with diltiazem (0.01–1000 μM), revealing lack of inhibition (Figure 2D, black line). As a positive control, an established GC pharmacological chaperone—N-(*n*-nonyl)deoxynojirimycin (NN-DNJ), exhibiting a half maximal inhibitory concentration (IC_50_) value of 1.08 μM toward Cerezyme (Figure 2D, blue line)—exhibited inhibition [14]. Collectively, these results demonstrate that diltiazem does not bind to the active site of GC ex vivo and is unlikely to function as a pharmacological chaperone.

To evaluate whether diltiazem could directly activate the existing lysosomal GC pool, L444P and N370S/V394L GC fibroblasts were incubated with diltiazem (1–100 μM) for 1 h, and the GC activity was measured using the intact cell assay. No activity increase was observed (Figure S3), demonstrating that the GC activity increase could not be achieved on this short time scale, a result inconsistent with direct diltiazem-induced, saposin-mediated activation of GC. A relatively long incubation period (5 d) is required to maximally increase intralysosomal L444P GC activity 2.0-fold (Figure 1C, left panel) and N370S/V394L GC activity 3.2-fold (Figure 1D, middle panel), suggesting that diltiazem likely requires new GC synthesis to achieve enhanced GC activity, consistent with the nearly identical rates of GC activity increases mediated by diltiazem treatment and pharmacological chaperone treatment [17].

### Diltiazem Does Not Influence GC Transcription

Quantitative reverse transcription (RT) PCR analysis was performed on L444P GC fibroblasts incubated without and with 10 μM diltiazem for 6 h, 12 h, 1 d, 3 d, and 5 d. Real-time PCR reactions were performed on total DNA reverse-transcribed from total RNA samples, which were extracted from L444P GC-harboring cells. The PCR amplification plot is shown in Figure 2E, left panel. ΔC_T_ is defined as the difference between the threshold cycle (C_T_) value of the GC gene and the C_T_ value of a housekeeping gene. The relative GC mRNA expression level was normalized to that of untreated GC cells, calculated from corresponding ΔC_T_ values (see Materials and Methods). No significant differences for the GC mRNA expression levels were observed when comparing untreated and diltiazem-treated L444P GC cells (Figure 2E, right panel, left entries) demonstrating that diltiazem does not influence GC transcription in L444P GC cells. Strictly analogous results were obtained for diltiazem-treated N370S/V394L GC cells (Figure 2E, right panel, right entries).

### Diltiazem Enhances Proper GC Folding and Trafficking

Cellular trafficking of L444P and N370S/V394L GC appears to be reduced because of ERAD outcompeting folding and trafficking [9]. Fluorescence microscopy was previously used to demonstrate that active-site–directed pharmacological chaperones enhance the folding and trafficking of G202R GC to the lysosome [10]. Strictly analogous immunofluorescence microscopy methods were used to demonstrate that Ca^2+^ channel blockers increase L444P and N370S/V394L GC trafficking to the lysosome. L444P GC harboring fibroblasts were cultured without or with 10 μM diltiazem for 14 d before plating for microscopy. WT GC fibroblasts were also studied analogously as a positive control. A properly folded and trafficked GC protein will colocalize with the lysosomal marker LAMP2 [10]. WT GC distributed in a punctate manner, and colocalized with LAMP2 (Figure 2F, column 3, row 3, GC in green, LAMP2 in red, and overlap artificially colored white). This color scheme is used only for the colocalization row; for single staining experiments (the first two rows), the fluorescence images are artificially colored white to improve contrast. While the L444P GC variant was not visible without diltiazem treatment, due to extensive ERAD, it was easily detected and was distributed in a punctate manner after diltiazem treatment (Figure 2G, column 1). L444P GC colocalized with LAMP2 after diltiazem treatment (Figure 2G, column 3, GC in green, LAMP2 in red, and overlap artificially colored white), indicating increased lysosomal trafficking, consistent with the increase in cellular GC concentrations (Figure 2A) and the increase in enzymatic activity (Figure 1C).

Previous experiments demonstrate that the N370S GC distribution is partially lysosomal [10]. To determine whether the increase in properly glycosylated N370S/V394L GC protein that was observed in response to diltiazem treatment (Figure 2C) resulted in an increase in proper trafficking to the lysosome, quantitative immunofluorescence microscopy was performed. N370S/V394L GC fibroblasts were incubated without and with 5 μM diltiazem for 7 d before plating for microscopy. WT GC fibroblasts served as a control. While measurable N370S/V394L GC colocalizes with the lysosome, there is substantially less N370S/V394L GC in the lysosome in comparison to WT GC (Figure 2F, compare column 2 with column 3), consistent with significant ERAD. Diltiazem treatment notably enhanced N370S/V394L GC trafficking to the lysosome (Figure 2F, compare column 1 with column 2), consistent with its ability to increase the concentration of the mature GC glycoform, Figure 2B and 2C. Quantification of the colocalization between the GC protein and the lysosomal marker using twenty random microscope fields for each sample was accomplished using Pearson's correlation coefficient (PCC). WT GC, untreated N370S/V394L GC, and diltiazem-treated N370S/V394L GC fibroblasts have PCC values of 0.70 ± 0.06, 0.60 ± 0.05, and 0.68 ± 0.05, respectively (Figure 2H). The difference between the PCC values of untreated and diltiazem-treated N370S/V394L GC cells is significant (*p* < 0.001, *n* = 20), demonstrating that diltiazem increased trafficking of N370S/V394L GC to the lysosome, nearly to WT levels.

### Extracellular Ca^2+^ Concentration Influences Intracellular Folding Capacity

Diltiazem and verapamil are both potent L-type voltage-gated calcium channel blockers that inhibit Ca^2+^ entry from the extracellular medium into the cell and thus alter calcium homeostasis in the cell [35]. The cytoplasmic free Ca^2+^ ion concentration (∼100 nM) is much lower than the extracellular Ca^2+^ ion concentration (∼2 mM) at steady state in a normal cellular environment. We explored whether manipulation of the extracellular Ca^2+^ concentration for prolonged periods could alter intracellular GC folding, trafficking and activity.

**Figure 3:**
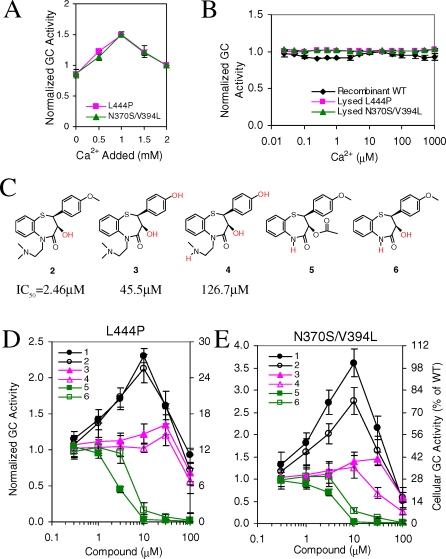
Intracellular Ca^2+^ Ion Concentration Influences GC Activity in L444P and N370S/V394L GC Fibroblasts (A) Variable Ca^2+^ ion cell culture media concentrations were applied to L444P GC cells for 10 d and to N370S/V394 GC cells for 7 d before using the lysed cell GC activity assay. The GC activity was normalized to that with 2 mM Ca^2+^ added in the media in both cases. (B) L444P and N370S/V394L GC cells were lysed and an equal amount of total cell protein was incubated with Ca^2+^ ions and their GC activities were evaluated using the lysed cell GC activity assay. Cerezyme, a recombinant WT GC enzyme, was also tested for its activity after Ca^2+^ treatment. (C) Chemical structure of diltiazem analogs (compounds **2**–**6**; distinct substructures relative to compound **1** are shown in red) with their reported Ca^2+^ channel blocker IC_50_ values [44]. (D) The influence of Ca^2+^ ion channel blockers of varying potency on L444P GC activity. L444P GC cells were incubated with compounds **1–6** for 7 d before using the intact cell GC activity assay to evaluate lysosomal GC activity. (E) The influence of Ca^2+^ ion channel blockers of varying potency on N370S/V394L GC activity. N370S/V394L GC cells were incubated with compounds **1–6** for 7 d before evaluating GC activity using the intact cell activity assay. For both (D) and (E), the GC activity of treated cells was normalized against that of untreated cells of the same type (left *y*-axis) and expressed as the percentage of WT GC activity (right *y*-axis).

Different Ca^2+^ ion concentrations (0, 0.5, 1, 1.5, and 2 mM CaCl_2_) added to Ca^2+^-free cell culture media (supplemented with fetal bovine serum) were applied to L444P GC cells for 10 d and to N370S/V394 GC cells for 7 d. The GC activity was then evaluated using the lysed cell GC activity assay. GC activity was normalized to that observed with 2 mM Ca^2+^ added in the media, similar to the concentration used in other experiments reported in this paper. The maximum GC activity increase (1.5-fold) was achieved when 1 mM Ca^2+^ was added to the media of L444P and N370S/V394L GC cells, demonstrating the important influence of extracellular Ca^2+^ ion concentration on GC folding and trafficking (Figure 3A).

Whether Ca^2+^ ions can interact directly with the GC protein was explored. Lysed L444P and N370S/V394L GC cells were incubated with variable Ca^2+^ ion concentrations (25 nM to 2 mM) and assayed using the lysed cell GC activity assay, indicating no significant changes in GC activity (Figure 3B). Cerezyme, a recombinant version of WT GC, was evaluated analogously, revealing unaltered activity (Figure 3B, black line). These results demonstrate that Ca^2+^ ions do not directly activate or inhibit the GC protein ex vivo*.*

### GC Activity Enhancement Correlates with Ca^2+^ Ion Channel Blocker Activity

To further examine the hypothesis that diltiazem enhances GC activity through its Ca^2+^ ion channel blocker activity, five diltiazem analogs exhibiting a range of potencies were procured (Figure 3C). The previously reported Ca^2+^ channel blocker IC_50_ values are: **1** (diltiazem, IC_50_ = 0.98 μM) > **2** (2.46 μM) > **3** (45.5 μM) > **4** (126.7 μM) [44]. Analogs **5** and **6** should not block Ca^2+^ ion channel activity because they lack a key basic amino nitrogen pharmacophore linked to N5 in the benzothiazepine ring scaffold, according to a reported structure-activity relationship (SAR) study on benzazepinone [45] and a quantitative SAR study on diltiazem [46].

L444P GC fibroblasts were cultured with compounds **1–6** (0.3–100 μM) for 7 d, and dose-response curves were recorded using the intact cell GC activity assay (Figure 3D). Compounds **1** (IC_50_ = 0.98 μM) and **2** (IC_50_ = 2.46 μM) are potent Ca^2+^ channel antagonists and exhibit notable L444P lysosomal GC activity increases to a maximum of 2.3-fold for **1** and 2.1-fold for **2** at 10 μM (Figure 3D, black lines). Compounds **3** (IC_50_ = 45.5 μM) and **4** (IC_50_ = 126.7 μM) are both weak Ca^2+^ channel antagonists and weak L444P GC activity enhancers. Notably, only at much higher concentrations (30 μM) do these low-potency analogs exhibit a maximum increase in L444P GC activity of 1.3-fold for **3** and 1.2-fold for **4** (Figure 3D, pink lines). Compounds **5** and **6** are not Ca^2+^ channel antagonists, and, as such, these closely related analogs do not increase L444P GC activity (Figure 3D, green lines). These data demonstrate that the more potent the Ca^2+^ ion channel blocker, the higher the lysosomal GC activity enhancement observed. Diltiazem and its analogs reveal analogous results in N370S/V394L fibroblasts (Figure 3E). Compound **1** (10 μM) increased N370S/V394L GC activity to a maximum of 3.6-fold, whereas compound **2** (10 μM) afforded a 2.8-fold increase, more than the increases observed with L444P cells. High concentrations of compounds **5** and **6** (>10 μM) are toxic to both L444P and N370S/V394L fibroblasts. At 100 μM, compounds **1–4** also lower GC activity due to cytotoxicity.

To further investigate the idea that diltiazem enhances GC activity by blocking plasma membrane Ca^2+^ ion channels, thus lowering intracellular Ca^2+^ concentrations, thapsigargin—a potent SERCA inhibitor—was applied to L444P GC cells without or with 10 μM diltiazem for 7 d. Thapsigargin inhibits Ca^2+^ entry into the ER from the cytosol, presumably leading to an increase in intracytoplasmic Ca^2+^ ion concentrations [33]. Therefore, diltiazem and thapsigargin have opposite effects on regulating cytosolic calcium homeostasis. Thapsigargin alone had no influence on GC activity until a concentration of 1 nM was reached; above this concentration, thapsigargin decreased L444P GC activity significantly after 7-d incubation (Figure S4, pink line). Co-application of varying concentrations of thapsigargin with 10 μM diltiazem revealed a thapsigargin dose-dependent decrease of GC activity (Figure S4, blue line), consistent with the hypothesis that these compounds have opposite influences on cytoplasmic Ca^2+^ ion levels and that lower rather than higher intracellular Ca^2+^ levels enhance mutant GC homeostasis.

### Diltiazem Treatment Up-Regulates the Expression of Chaperones

**Figure 4:**
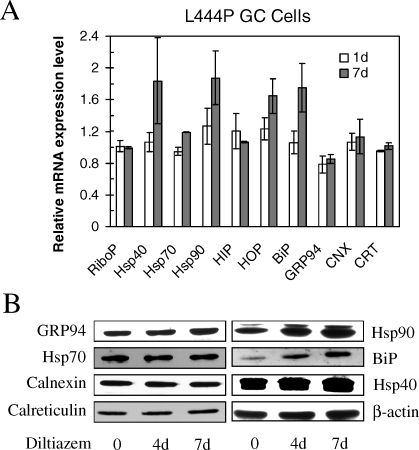
Chaperone Expression Level in Untreated and Diltiazem-Treated L444P GC Fibroblasts (A) Quantitative RT-PCR on untreated and diltiazem-treated L444P GC cells. L444P GC cells were incubated with 10 μM diltiazem for 1 d and 7 d. Relative mRNA expression level for diltiazem-treated L444P GC cells was normalized to that of untreated cells. Hsp40, Hsp70, Hsp90, HIP, HOP, BiP, GRP94, calnexin, and calreticulin were probed using corresponding primer pairs. Large ribosomal protein (RiboP) served as a housekeeping control. (B) L444P GC cells were treated with 10 μM diltiazem for 4 d and 7 d before being lysed for SDS-PAGE analysis. Hsp40, Hsp70, Hsp90, BiP, GRP94, calnexin, and calreticulin were probed using Western blot analysis. β-actin served as a loading control.

Molecular chaperones are known to be essential for the maintenance of cellular protein homeostasis [2,47–49]; hence it is possible that elevated chaperone expression levels could be responsible for the observed diltiazem-mediated enhancement in lysosomal enzyme homeostasis. Quantitative RT-PCR analysis was performed on L444P GC fibroblasts incubated without and with 10 μM diltiazem for 1 d and 7 d. The relative mRNA expression levels of representative cytoplasmic and ER lumenal chaperones—including Hsp40, Hsp70, BiP, Hsp90, GRP94, calnexin, calreticulin, HIP, and HOP—were probed and normalized to the levels found in untreated cells (Figure 4A). The large ribosomal protein (RiboP) was monitored as a control. All the primer pairs used are listed in Table S1. The mRNA expression levels of BiP, Hsp40, and Hsp90 were increased up to 1.8-fold, 1.8-fold, and 1.9-fold, respectively, after a 7-d treatment with diltiazem, whereas the mRNA expression levels of Hsp70, GRP94, calnexin, and calreticulin were not changed significantly. A strictly analogous RT-PCR analysis of N370S/V394L GC fibroblasts reveals similarly increased mRNA expression levels of Hsp40, however BiP and Hsp90 exhibit less of an increase after 7 d of diltiazem treatment (Figure S5). Western blot analysis was also performed on L444P GC fibroblasts incubated without and with diltiazem (10 μM) for 4 d and 7 d (Figure 4B). The increased protein expression levels of BiP, Hsp40, and Hsp90 were confirmed after a 7-d treatment with diltiazem. That the expression levels of GRP94, Hsp70, calnexin, and calreticulin were not changed significantly was also confirmed at the protein level. These increases in molecular chaperone expression levels, especially the cytoplasmic Hsp40 levels, seem to account for the increased GC folding capacity of the ER, and the requirement for new transcription may also contribute to the relatively slow increases in lysosomal enzyme levels upon calcium channel blocker treatment. Given the highly dynamic nature of the ER, it is envisioned that the cytosolic chaperones play a role in creating an ER optimized for protein folding and trafficking.

### Ca^2+^ Channel Blockers Improve Enzyme Homeostasis in Two Additional Lysosomal Storage Diseases Associated with Glycoprotein and Heparan Sulfate Accumulation

**Figure 5:**
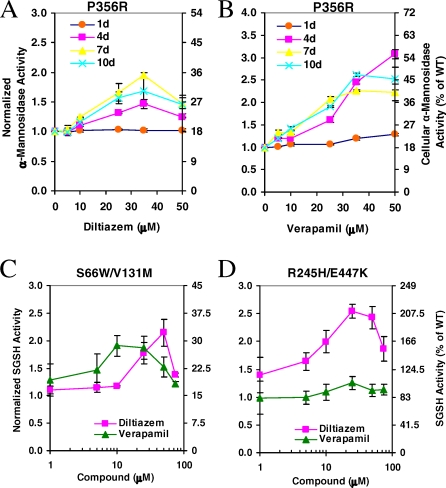
The Influence of Diltiazem and Verapamil on Mutant α-Mannosidase and Heparan Sulfate Sulfamidase (SGSH) Activity in Patient-Derived Fibroblasts The enzyme activity of treated cells was normalized against that of untreated cells of the same type (left *y*-axis) and expressed as the percentage of WT enzyme activity (right *y*-axis). (A) The influence of diltiazem on P356R α-mannosidase activity after culturing for 1 d (black line), 4 d (pink line), 7 d (blue line), and 10 d (yellow line). (B) The influence of verapamil on P356R α-mannosidase activity after culturing for 1 d (black line), 4 d (pink line), 7 d (blue line), and 10 d (yellow line). (C) The influence of diltiazem (pink line) and verapamil (green line) on S66W/V131M SGSH activity after culturing for 5 d. (D) The influence of diltiazem (pink line) and verapamil (green line) on R245H/E447K SGSH activity after culturing for 5 d, respectively. Unlike in Figure 1 and 3, percent activity relative to WT in Figure 5C and 5D is calculated from the specific activity of S66W and R245H reported in the literature [27].

Lysosomal α-mannosidase is a broad-specificity exoglycosidase involved in the ordered degradation of glycoproteins [24]. The P356R mutation in the α-mannosidase enzyme appears to compromise folding and trafficking, leading to very low lysosomal α-mannosidase activity and severe α-mannosidosis [26]. The activity of cells harboring P356R α-mannosidase is approximately 18% of that of WT α-mannosidase, under the assay conditions used. Incubation of these cells with a range of diltiazem or verapamil concentrations for 1, 4, 7, and 10 d enabled lysed cell enzyme activity analysis to be performed. Diltiazem (35 μM) increased the P356R α-mannosidase activity up to 2.0-fold after 7-d incubation period (≈36% the activity of WT α-mannosidase; Figure 5A). Verapamil (50 μM) increased the P356R α-mannosidase activity up to 3.1-fold (≈56% the activity of WT α-mannosidase) after an incubation period of 4 d (Figure 5B). Brief exposure of P356R α-mannosidase harboring cells to diltiazem or verapamil (1 d) did not increase α-mannosidase activity significantly (Figure 5A and 5B), indicating that it is likely that new protein synthesis is required for diltiazem and verapamil to affect cellular protein homeostasis, consistent with the result obtained from the Gaucher cell lines described above (Figure 1 and Figure S3).

The MPS type IIIA LSD is caused by a deficiency of SGSH, resulting in the defective degradation and storage of heparan sulfate, a glycosaminoglycan [25]. The common S66W and R245H mutations in type IIIA MPS lead to reduced specific activity (15% and 83% of normal specific activity for S66W and R245H, respectively) and lower cellular concentrations, likely a result of compromised folding and trafficking of the sulfamidase variants to the lysosome [27]. Two compound heterozygous MPS cell lines were used to evaluate the effect of diltiazem or verapamil, using an intact cell enzyme activity assay. In the case of the S66W/V131M MPS cells, diltiazem (50 μM) or verapamil (10 μM) treatment increased S66W/V131M sulfamidase activity up to 2.1-fold and 1.9-fold (≈30 % of WT sulfamidase activity), respectively, after a 5-d treatment, (Figure 5C). In the case of R245H/E447K MPS cells, diltiazem (25 μM) increased R245H/E447K SGSH activity up to 2.5-fold (≈207 % of WT sulfamidase activity), whereas verapamil did not change the sulfamidase activity significantly after 5-d treatment (Figure 5D).

## Discussion

We report the discovery that the L-type Ca^2+^ channel blockers diltiazem and verapamil restore partial folding, trafficking, and enzyme function to patient-derived fibroblasts in three distinct LSDs, disorders involving deficiencies in nonhomologous lysosomal enzymes that perform distinct chemical reactions. That these Ca^2+^ channel blockers are both FDA-approved drugs provides the incentive to conduct further necessary efficacy and safety experiments to discern whether they are promising candidates to ultimately treat neuropathic GD and related LSDs. Fortunately, diltiazem crosses the blood-brain barrier [50] and is bioavailable in the μM concentration range in blood plasma [51].

The Ca^2+^ ion channel blocker potency of diltiazem and its analogs correlates with their efficiency to enhance GC folding in the ER, enabling trafficking and the lysosomal localization of mutant GC in patient-derived fibroblast cell lines [52]. But how is the blockage of L-type Ca^2+^ channels on the plasma membrane by diltiazem coupled to enhanced mutant GC homeostasis? Activation of these channels allows extracellular Ca^2+^ to enter the cytosol, which subsequently induces further Ca^2+^ ion release from intracellular Ca^2+^ stores, such as the ER, by activating ryanodine receptors, the Ca^2+^ ion channels within the ER membrane. Inhibiting this calcium-induced calcium release [53] pathway minimizes depletion of the ER Ca^2+^ store, a process that appears to up-regulate the expression of a subset of cytosolic and ER chaperones, especially Hsp40. How blockage of L-type Ca^2+^ channels on the plasma membrane by diltiazem and verapamil influences gene transcription in the nucleus is unclear, and this signaling cascade merits further investigation.

Others have reported that the reduction in ER Ca^2+^ ion concentrations by SERCA inhibitors, such as curcumin [34] and thapsigargin [33], enhance folding and trafficking of ΔF508 CFTR. However, thapsigargin does not enhance L444P GC folding, trafficking, or lysosomal activity (Figure S4). Nor does diltiazem treatment increase the trafficking of ΔF508 CFTR to the plasma membrane (William E. Balch, personal communication). Diltiazem blocks calcium entry into the cytosol, whereas thapsigargin inhibits calcium movement from the cytosol into the ER. Therefore, diltiazem and thapsigargin regulate calcium homeostasis oppositely, presumably explaining why diltiazem and thapsigargin partially correct defective protein homeostasis in GD and cystic fibrosis, respectively.

Diltiazem is an FDA-approved small molecule used to treat angina and hypertension marketed under names including Cardizem, Dilacor, and Tiazec. Unlike pharmacological chaperones that directly bind to GC, thus stabilizing the folded enzyme in the ER for trafficking to the Golgi and on to the lysosome, diltiazem treatment of fibroblasts derived from patients with GD appears to alter the biological folding capacity of the ER. Diltiazem is well-tolerated, and the incidence of side effects is low. Its pharmacological properties have been extensively studied and reviewed [51,54,55]. Whereas diltiazem exhibited its best efficacy at increasing GC activity in patient-derived fibroblasts when used at a culture concentration of 10 μM, its lowest effective cell culture media concentration is in the range of 0.1–1 μM (Figures 2 and 3), equivalent to human plasma levels achieved by oral dosing.

### Summary

Diltiazem and verapamil, potent FDA-approved L-type Ca^2+^ channel blocker drugs, increased the ER folding capacity, trafficking, and activity of mutant lysosmal enzymes associated with three distinct LSDs. These compounds likely act through a Ca^2+^ ion mediated up-regulation of a subset of cytoplasmic and ER lumenal chaperones. Increasing ER calcium levels appears to be a relatively selective strategy to partially restore mutant lysosomal enzyme homeostasis in patient-derived cells, as ΔF508 CFTR folding efficiency and the folding efficiency of several other cellular WT enzymes was unaffected by these Ca^2+^ channel blockers.

## Materials and Methods

### Reagents.

Diltiazem hydrochloride (**1**) and verapamil were from Tocris Bioscience. Compound **2** was from Synfine. Compounds **3** and **4** were synthesized as described in Text S1. Ruthenium red, compounds **5** and **6**, 4-methylumbelliferyl β-D-glucopyranoside, 4-methylumbelliferyl α-D-mannopyranoside, 4-methylumbelliferyl α-D-glucopyranoside, 4-methylumbelliferyl β-D-galactopyranoside, 4-methylumbelliferyl α-D-galactopyranoside, and 4-methylumbelliferyl β-D-glucuronide were from Sigma. N-(*n*-nonyl)deoxynojirimycin (NN-DNJ), Conduritol B epoxide (CBE), and 4-methylumbelliferyl 2-sulfamino-2-deoxy-α-D-glucopyranoside were from Toronto Research Chemicals. All the other tested small molecules were either from Tocris Bioscience or from Sigma. Cell culture media were obtained from Gibco. Human injection-quality recombinant WT GC protein (trade name Cerezyme) was obtained from Genzyme.

### Cell cultures.

Primary skin fibroblast cultures were established from patients with GD who were homozygous for either the N370S GC (1226A>G) mutation or the G202R GC (721G>A) mutation. An apparently normal fibroblast (GM00498), three distinct homozygous Gaucher fibroblasts containing the L444P GC (1448T>C) mutation (GM08760, GM10915, and GM20272), two compound heterozygous Gaucher fibroblasts containing the N370S/V394L GC mutation (GM01607) and N370S/84GG GC mutation (GM00372), a homozygous α-mannosidosis fibroblast containing the P356R α-mannosidase mutation (GM04518), and two compound heterozygous type IIIA mucopolysaccharidosis fibroblasts containing the S66W/V131M SGSH mutation (GM01881) and R245H/E447K SGSH mutation (GM00879) were obtained from the Coriell Cell Repositories. Fibroblasts were maintained in minimum essential medium with Earle's salts supplemented with 10% heat-inactivated fetal bovine serum and 1% glutamine Pen-Strep at 37 °C in 5% CO_2_.

### Enzyme activity assays.

The intact cell GC activity assay has been previously described [14]. Briefly, cells were plated into 48-well assay plates (500 μl per well). After cell attachment, the media was replaced by media containing small molecules. Media was changed every 3 d. After incubation at 37 °C for the indicated amount of time, the intact cell GC activity assay was performed. The monolayers were washed by DPBS. The reaction was started by the addition of 150 μl of 3 mM 4-methylumbelliferyl β-D-glucopyranoside in 0.2 M acetate buffer (pH 4.0) to each well, followed by incubation at 37 °C for 1–7 h. CBE was used as a control to evaluate the extent of nonspecific GC activity. The reaction was stopped by lysing the cells with 750 μl of 0.2 M glycine buffer (pH 10.8). Liberated 4-methylumbelliferone was measured (excitation 365 nm, emission 445 nm) with a SpectraMax Gemini plate reader (Molecular Devices). The lysed cell GC activity assay has been previously described [14]. Briefly, intact cells were harvested and the pellet was lysed in the complete lysis-M buffer containing complete protease inhibitor cocktails (Roche #10799050001). Total cell protein was measured using the Micro BCA assay reagent (Pierce #23235). Thirty μg of total cell protein was assayed for the GC activity in 100 μl of 0.1 M acetate buffer (pH 5.0) containing 3 mM 4-methylumbelliferyl β-D-glucopyranoside in the presence of 0.15% Triton X-100 (v/v, Fisher) and 0.15% taurodeoxycholate (w/v, Calbiochem). CBE was used as a control to evaluate the extent of nonspecific GC activity. After incubation at 37 °C for 1–7 h, the reaction was terminated with 200 μl of 0.2 M glycine buffer (pH 10.8), and the fluorescence was recorded (excitation 365 nm, emission 445 nm). The GC activity assay for recombinant WT GC enzymes has been previously described [10]. 25 ng of recombinant WT GC protein was assayed for the GC activity in 50 μl of 0.1 M acetate buffer (pH 5.0) containing 3 mM 4-methylumbelliferyl β-D-glucopyranoside in the presence of 0.15% Triton X-100 (v/v, Fisher) and 0.15% taurodeoxycholate (w/v, Calbiochem). After the addition of tested compounds, the reaction was incubated at 37 °C for 20 min, terminated with 75 μl of 0.2 M glycine buffer (pH 10.8), and the fluorescence was recorded (excitation 365 nm, emission 445 nm).

The activity of lysosomal α-mannosidase was determined as previously described with minor modification by using 2 mM 4-methylumbelliferyl α-D-mannopyranoside as the substrate [26]. The activity of lysosomal SGSH was determined by using 0.5 mM 4-methylumbelliferyl 2-sulfamino-2-deoxy-α-D-glucopyranoside as previously described with minor modification [56]. The activities of lysosomal enzymes α-glucosidase, β-galactosidase, α-galactosidase, and β-glucuronidase were assayed as previously described by using corresponding substrates 4-methylumbelliferyl α-D-glucopyranoside, 4-methylumbelliferyl β-D-galactopyranoside, 4-methylumbelliferyl α-D-galactopyranoside, and 4-methylumbelliferyl β-D-glucuronide, respectively [17].

Small molecules were evaluated at least in triplicate at each concentration, and each molecule was assayed at least three times. The data reported were normalized to the enzyme activity of untreated cells of the same type and expressed as percentage of WT enzyme activity.

### Quantitative RT-PCR.

The cells were incubated with 10 μM diltiazem at 37 °C for the indicated amount of time. Total RNA was extracted from the cells using RNeasy Mini Kit (Qiagen #74104). cDNA was synthesized from 500 ng of total RNA using QuantiTect Reverse Transcription Kit (Qiagen #205311). Quantitative PCR reactions were performed using QuantiTect SYBR Green PCR Kit (Qiagen #204143) and corresponding primers in the ABI PRISM 7900 system (Applied Biosystems). The forward and reverse primers for GC, Hsp40, Hsp70, Hsp90, HIP, HOP, BiP, GRP94, calnexin (CNX), and calreticulin (CRT), and an endogenous housekeeping gene large ribosomal protein (RiboP) are listed in Table S1. Samples were heated for 15 min at 95 °C and amplified in 45 cycles of 15 s at 94 °C, 30 s at 59 °C, and 30 s at 72 °C. Analysis was done using SDS2.1 software (Applied Biosystems). Threshold cycle (C_T_) was extracted from the PCR amplification plot. The ΔC_T_ value was used to describe the difference between the C_T_ of a target gene and the C_T_ of the housekeeping gene: ΔC_T_ = C_T_ (target gene) – C_T_ (housekeeping gene). The relative mRNA expression level of a target gene of diltiazem-treated cells was normalized to that of untreated cells: Relative mRNA expression level = 2 exp [–(ΔC_T_ (treated cells) – ΔC_T_ (untreated cells))].

### Western blot.

The cells were lysed using the complete lysis-M buffer containing complete protease inhibitor cocktails (Roche #10799050001). Total cell protein was measured using the Micro BCA assay reagent. Endo H (New England Biolabs #P0703) was used to digest the cell lysates according to the company instructions. The cell lysates containing equal amount of total protein were separated by 10% SDS-PAGE. Western blot analysis was performed with proper antibodies. Mouse monoclonal anti-GC 8E4 [57] was kindly provided by Klaus-Peter Zimmer (Children's Hospital of the University of Münster, Münster, Germany). Mouse monoclonal anti-GC 2E2 was from Novus Biologicals (#H00002629-M01). Antibodies directed against calnexin (#SPA-860), calreticulin (#SPA-601), Hsp40 (#SPA-400), Hsp70 (#SPA-812), and Hsp90 (#SPA-830) were from Stressgen. Antibodies directed against BiP (#SC-13968) and GRP94 (#SC-11402) were from Santa Cruz Biotechnology. Mouse monoclonal anti-β actin AC-15 was from Sigma (#A1978). Secondary antibodies (#31430 for goat anti-mouse and #31460 for goat anti-rabbit) were from Pierce. Bands were visualized using the SuperSignal West Pico Chemiluminescent Substrate (Pierce #34078) or SuperSignal West Femto Maximum Sensitivity Substrate (Pierce #34095).

### Immunofluorescence.

Immunofluorescence has been previously described [10]. Cells grown on glass cover slips were washed by PBS and fixed with 3.7% paraformaldehyde in PBS for 15 min. The slips were washed with PBS, quenched with 15 mM glycine in PBS for 10 min, and permeabilized with 0.2% saponin in PBS for 15 min. The antibodies were prepared in PBS in the presence of 0.2% saponin and 5 % goat serum. Cells were incubated for 1 h with primary antibodies (1:100 for mouse monoclonal anti-GC 16B3 [58] (kindly provided by Ernest Beutler, The Scripps Research Institute, La Jolla, CA), or 1:100 for 8E4, and 1:10,000 for rabbit anti-LAMP2 [59] (kindly provided by Michiko Fukuda, The Burnham Institute, La Jolla, CA)), washed with 5 % goat serum in PBS, and then incubated for 1 h with secondary antibodies (Alexa Fluor 488 goat anti-mouse IgG (#A11029) and Alexa Fluor 546 goat anti-rabbit IgG (#A11035)) from Molecular Probes. The cover slips were mounted and sealed. Images were collected using a Bio-Rad (Zeiss) Radiance 2100 Rainbow laser scanning confocal microscope attached to a Nikon TE2000-U microscope. For quantitative colocalization analysis, Z-stacks of each frame were flattened, and PCC was calculated using NIH Image J software. Random frames from each slide were averaged and colocalization differences were analyzed using a two-tailed Student's *t*-test.

## Supporting Information

Figure S1 The Influence of Ruthenium Red on L444P GC Activity in Gaucher Patient-Derived Fibroblasts after Culturing for 1 d (Black Line), 3 d (Pink Line), and 5 d (Green Line) The GC activity of treated cells was normalized against that of untreated L444P GC cells (left *y*-axis) and expressed as the percentage of WT GC activity (right *y*-axis).
(42 KB PDF)

Figure S2 The Influence of Dilitazem on the Activity of Lysosomal Enzymes After incubation with 10 μM diltiazem for 5 d, WT fibroblasts were assayed for the activities of GC, α-mannosidase, α-glucosidase, and β-galactosidase using intact cell enzyme activity assay, and L444P GC cells were assayed for the activities of GC, α-mannosidase, α-glucosidase, β-galactosidase, α-galactosidase, and β-glucuronidase using lysed cell enzyme activity assay. The enzyme activity of treated cells was normalized against that of untreated cells of the same type.
(45 KB PDF)

Figure S3 L444P and N370S/V394L GC Cells Were Incubated with Diltiazem for 1 h, and Their GC Activities Were Evaluated Using the Intact Cell GC Activity Assay The GC activity of treated cells was normalized against that of untreated cells of the same type.
(40 KB PDF)

Figure S4 The Influence of Thapsigargin and Diltiazem on GC Activity in L444P GC Fibroblasts Thapsigargin was applied without or with 10 μM diltiazem for 7 d. The GC activity of treated cells was normalized against that of untreated L444P GC cells (left *y*-axis) and expressed as the percentage of WT GC activity (right *y*-axis).
(45 KB PDF)

Figure S5 Quantitative RT-PCR Analysis on Untreated and Diltiazem-Treated N370S/V394L GC Cells N370S/V394L GC cells were incubated with 10 μM diltiazem for 1 d and 7 d, respectively. Relative mRNA expression level for diltiazem-treated N370S/V394L GC cells was normalized to that of untreated cells. Hsp40, Hsp70, Hsp90, HIP, HOP, BiP, GRP94, calnexin (CNX), and calreticulin (CRT) were probed using corresponding primer pairs. Large ribosomal protein (RiboP) served as a housekeeping gene control.
(35 KB PDF)

Table S1 Primer Sequences Used in Quantitative RT-PCR (46 KB DOC)

Text S1 Syntheses and Structural Characterization of Compounds **3** and **4**
(61 KB DOC)

## Abbreviations

CFTR: cystic fibrosis transmembrane conductance regulator
CNS: central nervous system
ER: endoplasmic reticulum
ERAD: endoplasmic reticulum–associated degradation
GC: glucocerebrosidase
GD: Gaucher disease
LSD: lysosomal storage disease
MPS: mucopolysaccharidosis
PCC: Pearson's correlation coefficient
SERCA: sarcoplasmic endoplasmic reticulum Ca^2+^-ATPase
SGSH: heparan sulfate sulfamidase
TRP: transient receptor potential
VGCC: voltage-gated calcium channels
WT: wild type
