# Supplementary material for: Partial Restoration of Mutant Enzyme Homeostasis in Three Distinct Lysosomal Storage Disease Cell Lines by Altering Calcium Homeostasis
Source: PLoS Biol. 2008 Feb 5;6(2):e26. doi: 10.1371/journal.pbio.0060026 (PMC2225441; doi:10.1371/journal.pbio.0060026)
Supplement: Supplementary file 1 [file pbio.0060026.sg001.pdf]

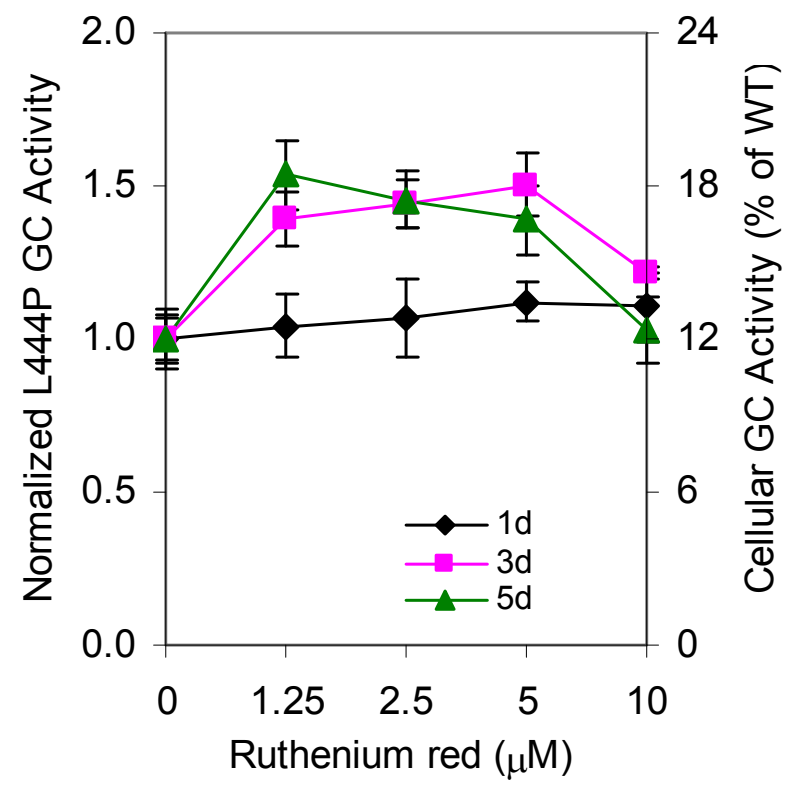
Normalized L444P GC Activity
Cellular GC Activity (% of WT)
Ruthenium red (μM)
2.0
1.5
1.0
0.5
0.0
24
18
12
6
0
0
1.25
2.5
5
10
1d
3d
5d

Figure S1
