# Supplementary material for: Partial Restoration of Mutant Enzyme Homeostasis in Three Distinct Lysosomal Storage Disease Cell Lines by Altering Calcium Homeostasis
Source: PLoS Biol. 2008 Feb 5;6(2):e26. doi: 10.1371/journal.pbio.0060026 (PMC2225441; doi:10.1371/journal.pbio.0060026)
Supplement: Supplementary file 2 [file pbio.0060026.sg002.pdf]

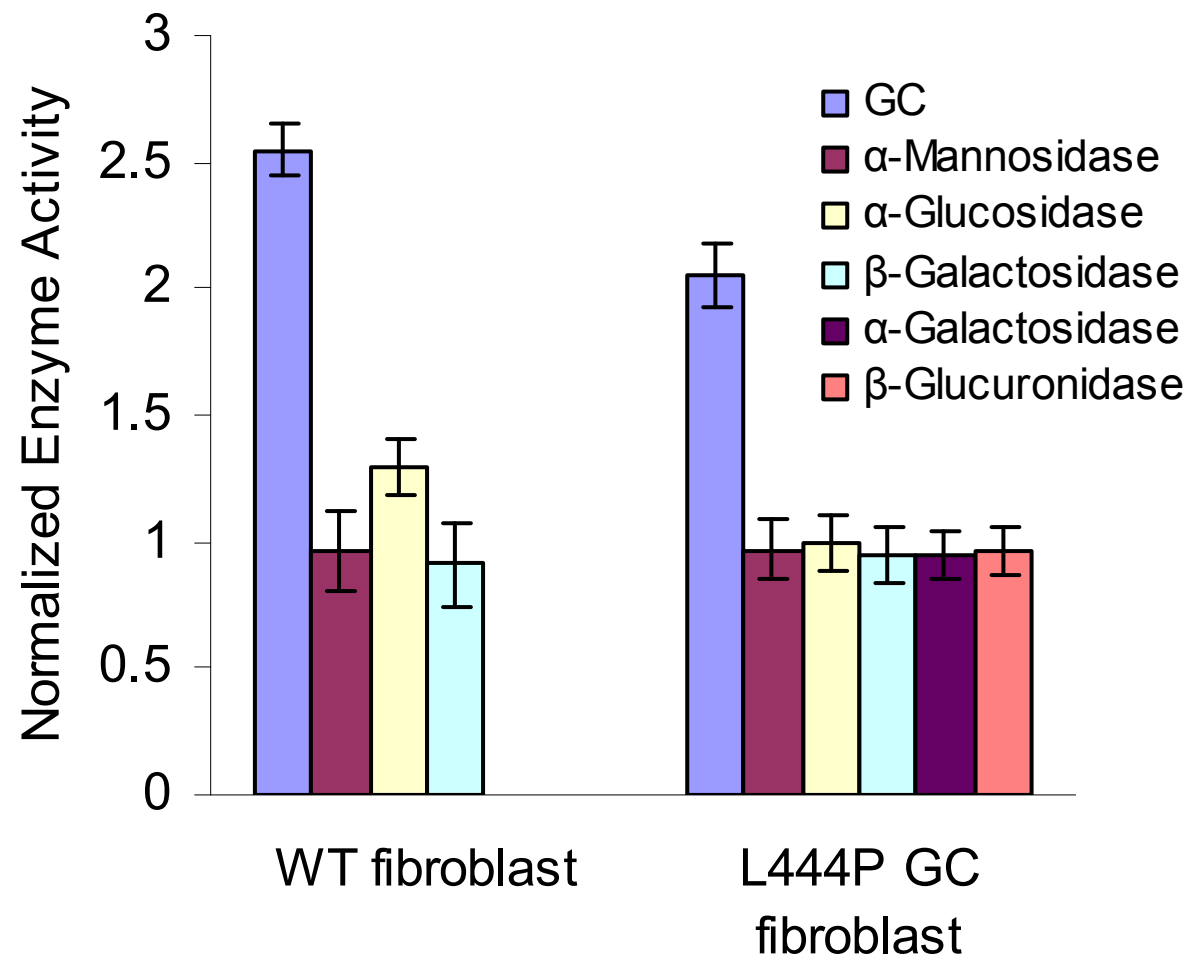
3
2.5
2
1.5
1
0.5
0
Normalized Enzyme Activity
GC
α-Mannosidase
α-Glucosidase
β-Galactosidase
α-Galactosidase
β-Glucuronidase
WT fibroblast
L444P GC fibroblast

Figure S2
