# Supplementary material for: Partial Restoration of Mutant Enzyme Homeostasis in Three Distinct Lysosomal Storage Disease Cell Lines by Altering Calcium Homeostasis
Source: PLoS Biol. 2008 Feb 5;6(2):e26. doi: 10.1371/journal.pbio.0060026 (PMC2225441; doi:10.1371/journal.pbio.0060026)
Supplement: Supplementary file 3 [file pbio.0060026.sg003.pdf]

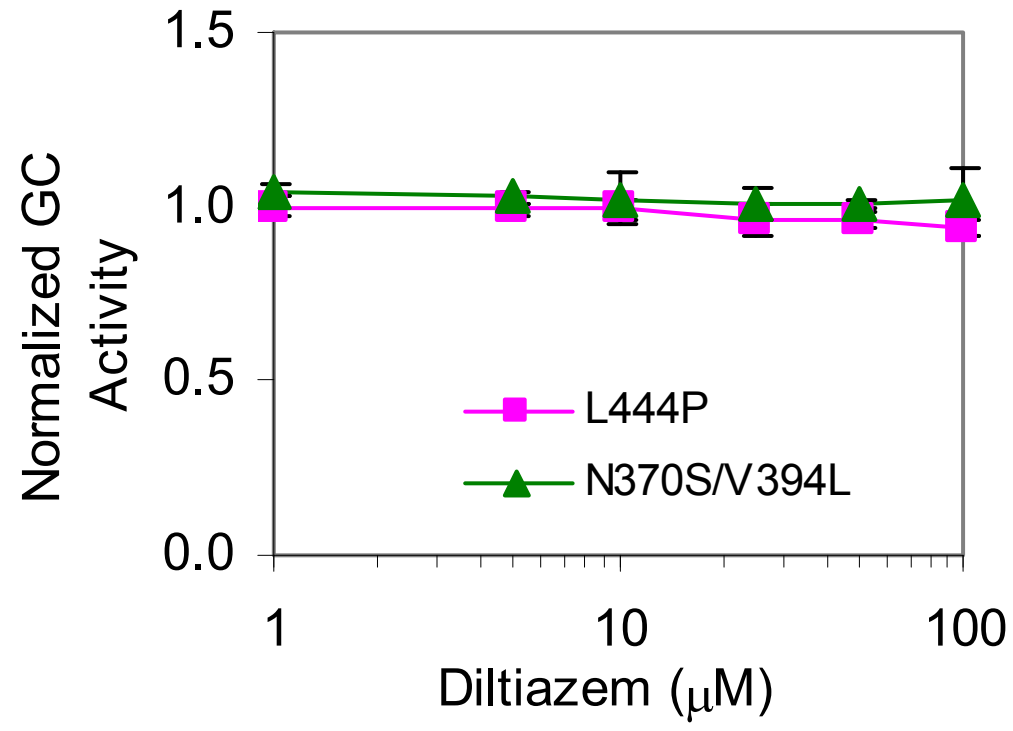

1.5
1.0
0.5
0.0
Normalized GC Activity
L444P
N370S/V394L
1
10
100
Diltiazem (μM)

Figure S3
