# Supplementary material for: Partial Restoration of Mutant Enzyme Homeostasis in Three Distinct Lysosomal Storage Disease Cell Lines by Altering Calcium Homeostasis
Source: PLoS Biol. 2008 Feb 5;6(2):e26. doi: 10.1371/journal.pbio.0060026 (PMC2225441; doi:10.1371/journal.pbio.0060026)
Supplement: Supplementary file 4 [file pbio.0060026.sg004.pdf]

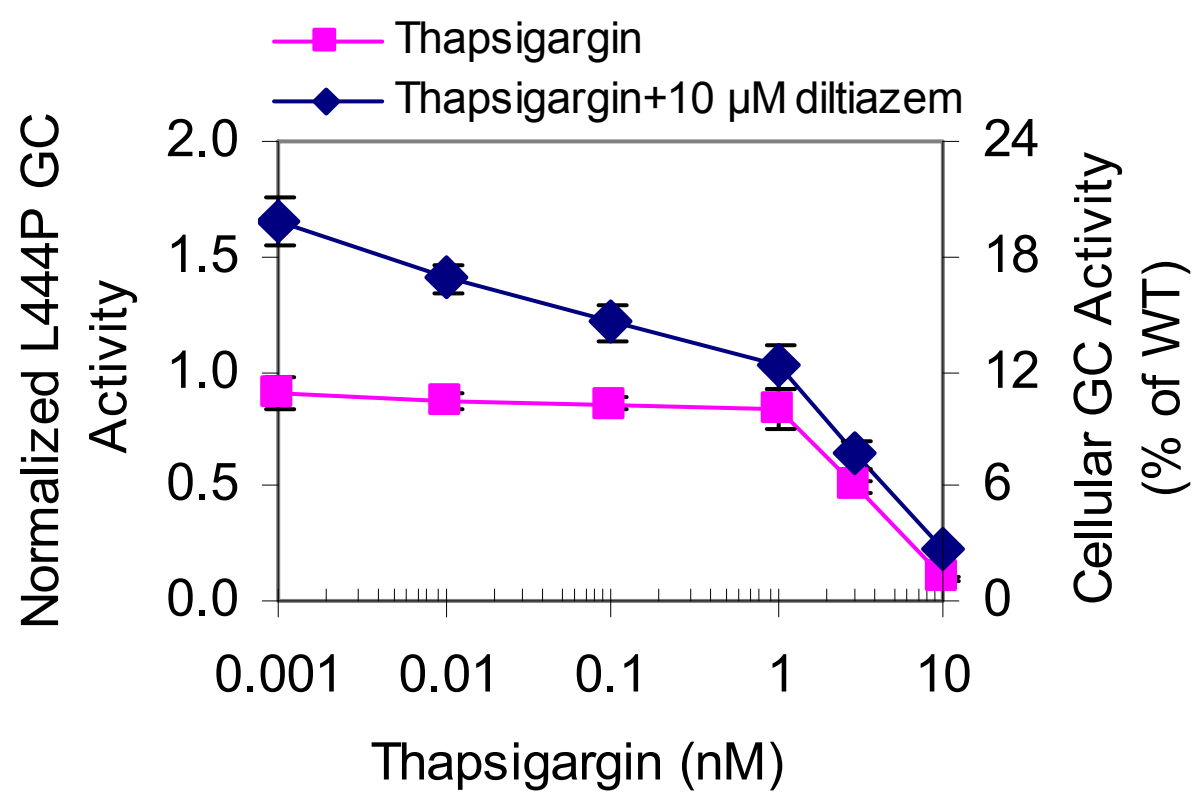

Thapsigargin
Thapsigargin+10 μM diltiazem
Normalized L444P GC Activity
Cellular GC Activity (% of WT)
2.0
1.5
1.0
0.5
0.0
24
18
12
6
0
0.001
0.01
0.1
1
10
Thapsigargin (nM)

Figure S4
