# Supplementary material for: Partial Restoration of Mutant Enzyme Homeostasis in Three Distinct Lysosomal Storage Disease Cell Lines by Altering Calcium Homeostasis
Source: PLoS Biol. 2008 Feb 5;6(2):e26. doi: 10.1371/journal.pbio.0060026 (PMC2225441; doi:10.1371/journal.pbio.0060026)
Supplement: Supplementary file 5 [file pbio.0060026.sg005.pdf]

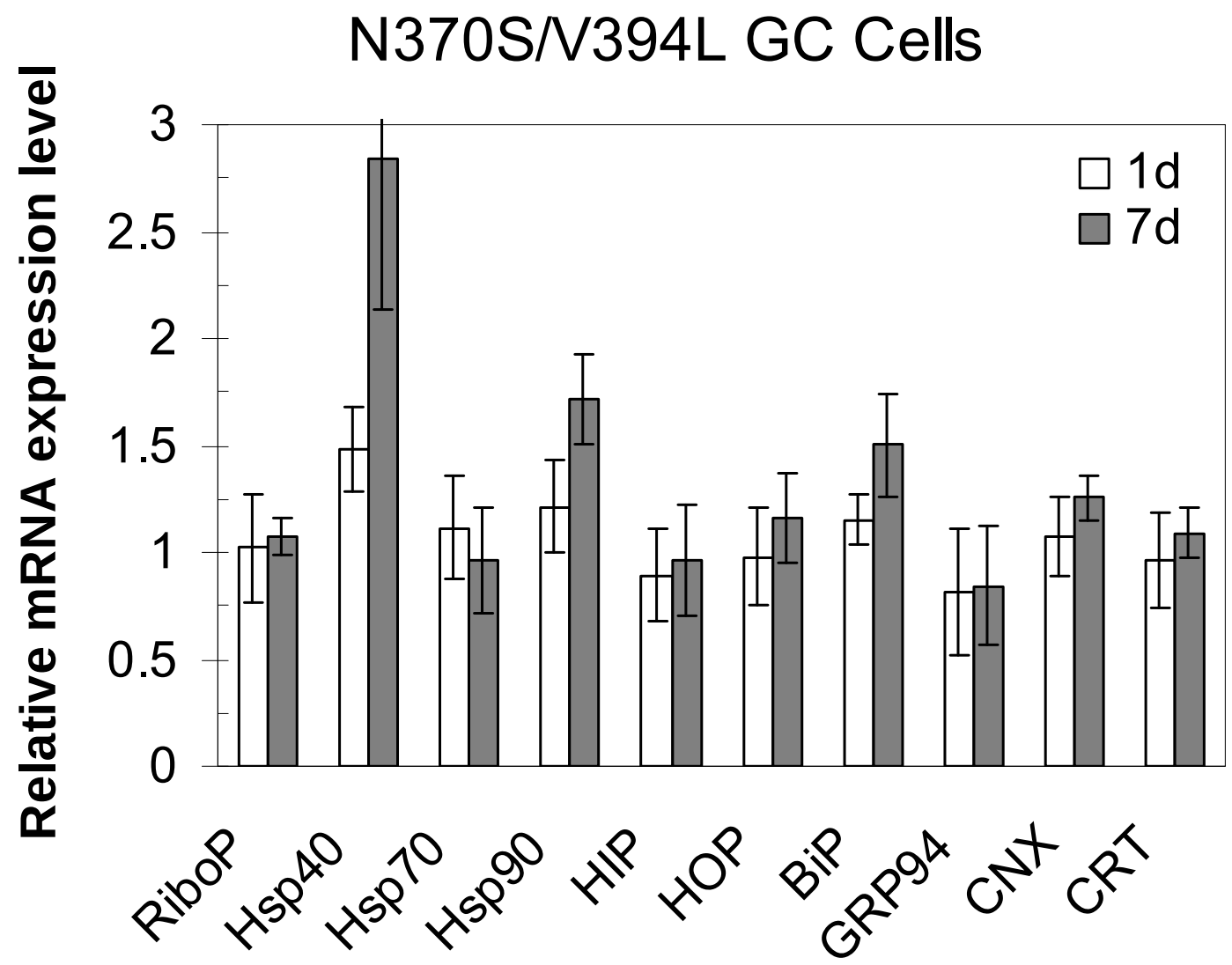
N370S/V394L GC Cells
Relative mRNA expression level
3
2.5
2
1.5
1
0.5
0
1d
7d
RiboP
Hsp40
Hsp70
Hsp90
HIP
HOP
BiP
GRP94
CNX
CRT

Figure S5
