# Supplementary material for: Partial Restoration of Mutant Enzyme Homeostasis in Three Distinct Lysosomal Storage Disease Cell Lines by Altering Calcium Homeostasis
Source: PLoS Biol. 2008 Feb 5;6(2):e26. doi: 10.1371/journal.pbio.0060026 (PMC2225441; doi:10.1371/journal.pbio.0060026)
Supplement: Supplementary file 6 [file pbio.0060026.sd001.doc]

**Supporting Text S1**

**Syntheses and structural characterization of compounds 3 and 4**

Reagents were purchased from Aldrich. NMR spectra were recorded on a Varian FT NMR spectrometer at a proton frequency of 400 MHz. High-resolution mass spectra (HRMS) were obtained at The Scripps Research Institute Center for Mass Spectrometry. High performance liquid chromatography (HPLC) separations were performed on a Waters dual 600 pump liquid chromatography system equipped with a Waters 2487 PDA (photodiode array) UV detector using a Phenomenex Jupiter 4u Proteo 90A reverse phase C18 column (250x21.20 mm) for preparative HPLC.

**Scheme 1.** Synthesis of compound **3**. Reagents: (a) KOH, EtOH (quantitative);

(b) SiCl_4_/LiI/BF_3_⋅OEt_2_, acetonitrile/toluene, 70 °C (81%).

Although the syntheses of compounds **3** and **4** were reported previously [1,2], they either required multiple steps or gave low overall yields. Here we utilized inexpensive commercially available compounds as the starting materials to obtain compounds **3** and **4** in two steps with good yields, respectively. The synthetic route of compound **3** is shown in Scheme 1. Compound **2** was prepared from commercially available diltiazem hydrochloride in quantitative yield as previously reported [3]. Compound **3** was achieved by O-demethylation of **2** in 81% yield with SiCl_4_/LiI in the presence of a catalytic amount of BF_3_ [4]. The synthetic route of compound **4** is shown in Scheme 2. Compound **7** was prepared from commercially available compound **6** in 71% yield as previously reported [1]. Compound **4** was obtained by both O-demethylation and the removal of the Cbz protecting group from compound **7** in one pot using BBr_3_ in 61% yield [5,6].

**Scheme 2.** Synthesis of compound **4**. Reagents: (a) NaH/DMSO, ClCH_2_CH_2_NMeCbz (71%);

(b) BBr_3_, CH_2_Cl_2_, -18 °C to RT (61%).

**(*2S,3S*)-5-[2-(Dimethylamino)ethyl]-2,3-dihydro-3-hydroxy-2-(4-hydroxyphenyl)-1,5-benzothiazepin-4(*5H*)-one (3).** 432 mg of **2** (1.16 mmol) was dissolved in 15 ml of anhydrous toluene. 1553 mg of LiI (11.6 mmol) and 5 ml of acetonitrile were added followed by 11.6 ml of 1M SiCl_4_ in CH_2_Cl_2_ (11.6 mmol) and 294 μl of BF_3_⋅OEt_2_ (2.32 mmol). The mixture was stirred for 16 h at 70 °C. The reaction was quenched by the addition of 25 ml of methanol and excessive solid Na_2_CO_3_, filtered, and concentrated. The mixture was re-dissolved in 25 ml of CHCl_3_ and 25 ml of H_2_O. The pH value was adjusted to 9.0 with saturated Na_2_CO_3_ solution. The mixture was extracted with CHCl_3_ (15 ml x 3). The combined organic layer was dried over Na_2_SO_4_, and rotary-evaporated. Flash chromatography (1:9 methanol/CH_2_Cl_2_) gave 337 mg (81%) of **3** as a pale-yellow powder. ^1^H NMR (400MHz, d_6_-DMSO) δ = 2.16 (s, 6H), 2.28-2.34 (m, 1H), 2.56-2.61 (m, 1H), 3.67-3.74 (m, 1H), 4.27-4.35 (m, 1H), 4.17 (t, *J* = 7.1Hz, 1H), 4.41 (d, *J* = 7.4Hz, 1H), 4.82 (d, *J* = 7.3Hz, 1H), 6.69-7.67 (m, 8H), 9.43 (s, 1H); ^13^C NMR (100MHz, d_6_-DMSO) δ = 45.09, 46.55, 56.05, 56.40, 68.50, 114.50, 124.57, 125.38, 127.11, 128.15, 130.58, 131.17, 134.56, 145.13, 157.17, 170.62; HRMS for C_19_H_22_N_2_O_3_S [M+H]^+^ calc, 359.1424; found, 359.1427.

**(*2S,3S*)-2,3-Dihydro-3-hydroxy-2-(4-hydroxyphenyl)-5-[2-(methylamino)ethyl]-1,5-benzothiazepin-4(*5H*)-one (4).** 138 mg of **7** (0.28 mmol) in 5 ml of anhydrous CH_2_Cl_2_ was cooled to -18 °C. 2 ml of 1M BBr_3_ in CH_2_Cl_2_ (2 mmol) was added dropwise. The reaction was stirred at -18 °C for 1 h and then at room temperature for another 10 h. 10 ml of H_2_O was added to the mixture dropwise. The pH value was adjusted to 9.0 with NaOH solution. The mixture was extracted with ethyl acetate (20 ml x 3). The combined organic layer was dried over Na_2_SO_4_, and rotary-evaporated. The crude product was purified by preparative HPLC using a reverse phase C18 column to give 78 mg (61%) of the CF_3_COOH salt of **4** as a white powder. ^1^H NMR (400MHz, D_2_O) δ = 2.76 (s, 3H), 3.28-3.35 (m, 1H), 3.43-3.49 (m, 1H), 4.06-4.13 (m, 1H), 4.43-4.50 (m, 1H), 4.52 (d, *J* = 7.6Hz, 1H), 4.96 (d, *J* = 7.6Hz, 1H), 6.90-7.76 (m, 8H); ^13^C NMR (100MHz, D_2_O) δ = 33.22, 45.61, 46.85, 55.87, 69.45, 115.46, 124.74, 126.03, 127.86, 128.95, 131.52, 131.60, 135.35, 143.68, 156.18, 173.55; HRMS for C_18_H_20_N_2_O_3_S [M+H]^+^ calc, 345.1267; found, 345.1279.

**References**

1. Miyazaki M, Iwakuma T, Tanaka T (1978) Synthesis of metabolites and related compounds of diltiazem. Chem Pharm Bull 26: 2889-2893.

2. Li RS, Farmer PS, Xie M, Quilliam MA, Pleasance S, et al. (1992) Synthesis, Characterization, and Ca^2+^ antagonistic activity of diltiazem metabolites. J Med Chem 35: 3246-3253.

3. Maiorana S, Brocchetti DG, Piacenza G (1985) 1,5-Benzothiazepines with cardiovascular utility, and pharmaceutical compositions containing them. US 4547495.

4. Zewge D, King A, Weissman S, Tschaen D (2004) Enhanced O-dealkylation activity of SiCl_4_/LiI with catalytic amount of BF_3_. Tetrahedron Lett 45: 3729-3732.

5. McOmie JFW, Watts ML, West DE (1968) Demethylation of aryl methyl ethers by boron tribromide. Tetrahedron 24: 2289-2292.

6. Felix AM (1974) Cleavage of protecting group with boron tribromide. J Org Chem 39: 1427-1429.
