# Supplementary material for: Partial Restoration of Mutant Enzyme Homeostasis in Three Distinct Lysosomal Storage Disease Cell Lines by Altering Calcium Homeostasis
Source: PLoS Biol. 2008 Feb 5;6(2):e26. doi: 10.1371/journal.pbio.0060026 (PMC2225441; doi:10.1371/journal.pbio.0060026)
Supplement: Supplementary file 7 [file pbio.0060026.st001.doc]

**Table S1.** Primer sequences used in quantitative RT-PCR

| Gene | GenBank  Accession code | Forward Primer | Reverse Primer |
| --- | --- | --- | --- |
| GC | M16328 | 5’-CTC CAT CCG CAC CTA CAC C-3’ | 5’-ATC AGG GGT ATC TTG AGC TTG G-3’ |
| RiboP | NM_001004 | 5’-CGT CGC CTC CTA CCT GCT-3’ | 5’-CCA TTC AGC TCA CTG ATA ACC TTG-3’ |
| Hsp40 | NM_006145 | 5'-CGC CGA GGA GAA GTT C-3' | 5'-CAT CAA TGT CCA TGC CTT-3' |
| Hsp70 | NM_005345 | 5'-GGA GGC GGA GAA GTA CA-3' | 5'-GCT GAT GAT GGG GTT ACA-3' |
| Hsp90 | NM_005348 | 5'-GAT AAA CCC TGA CCA TTC C-3' | 5'-AAG ACA GGA GCG CAG TTT CAT AAA-3' |
| HIP | NM_003932 | 5'-CCG CAA AGT GAA CGA G-3' | 5'-TGA TGG TTC GTC TGC C-3' |
| HOP | NM_006819 | 5'-ATG ACC ACT CTC AGC GTC-3' | 5'-CTC CTT GGC TTT GTC GTA-3' |
| BiP | NM_005347 | 5'-GCC TGT ATT TCT AGA CCT GCC-3' | 5'-TTC ATC TTG CCA GCC AGT TG-3' |
| GRP94 | NM_003299 | 5'-GGC CAG TTT GGT GTC GGT TT-3' | 5'-CGT TCC CCG TCC TAG AGT GTT-3' |
| CNX | NM_001746 | 5'-GCG TTG TGG GGC AGA TGA T-3' | 5'-CCG GTT GAG GTG CAT CAG T-3' |
| CRT | NM_004343 | 5'-AAG TTC TAC GGT GAC GAG GAG-3' | 5'-GTC GAT GTT CTG CTC ATG TTT C-3' |
